# Frailty as a novel predictor of achieving comprehensive disease control (CDC) in rheumatoid arthritis

**DOI:** 10.1007/s10067-021-05744-1

**Published:** 2021-07-20

**Authors:** Fausto Salaffi, Rossella De Angelis, Sonia Farah, Marina Carotti, Marco Di Carlo

**Affiliations:** 1 Rheumatology Clinic, Università Politecnica delle Marche, Ospedale “Carlo Urbani”, Via Aldo Moro, 25, 60035, Jesi, Ancona, Italy; 2 Radiology Department, Università Politecnica delle Marche, Ospedali Riuniti, Ancona, Italy

**Keywords:** Comprehensive disease control, Frailty, Outcome measures, Rheumatoid arthritis

## Abstract

**Background:**

Frailty is a construct recently introduced in the context of inflammatory joint diseases. To date, it is not clear if frailty can act as a negative factor in the achievement of comprehensive disease control (CDC) in patients suffering from rheumatoid arthritis (RA).

**Aim:**

To verify whether frailty is a factor hindering the achievement of CDC in patients with RA starting a biologic drug.

**Methods:**

RA patients requiring a treatment with a biologic drug were included. Patients were classified as achieving or not achieving CDC after 12 months of treatment. Patients were classified as non-frail, mildly frail, moderately frail and severely frail according to the Comprehensive Rheumatologic Assessment of Frailty (CRAF). Frailty was tested using the Mann–Whitney or Kruskal-Wallis test for continuous variables and chi-square test or Fisher’s exact test for comparison with categorical variables. A multivariable logistic regression was performed to identify factors associated with prediction of CDC achievers.

**Results:**

A total of 214 RA patients were followed for 12 months, 14.5% achieved CDC. Eighty-four (39.3%) patients were non-frail, 57 (26.6%) were mildly frail, 14 (6.5%) were moderately frail and 59 (27.6%) were severely frail. The multivariable logistic regression analysis identified the CRAF score at baseline as an independent variable for CDC achievement at 12 months (*p* = 0.0040).

**Discussion:**

Frailty is a frequent condition in RA patients and reduces the chances of achieving CDC.

**Conclusions:**

Frailty, measured by CRAF, reduced the likelihood of CDC achievement in RA patients treated with a biologic agent.

**Table:** 

Key Points • *Frailty is an under-researched condition in rheumatoid arthritis affecting more than 60% of patients.* • *Frailty is a condition that hinders the achievement of comprehensive disease control after 1 year of treatment with biological drugs in patients with rheumatoid arthritis.*

## Introduction

Rheumatoid arthritis (RA) is an inflammatory autoimmune joint disease that can lead to a significant disability and poor quality of life [1]. RA is, in a certain way, considered an immunological “emergency”, and the introduction at an early stage of a treatment with disease-modifying anti-rheumatic drugs (DMARD) is essential to suppress inflammation, maintain normal joint structure and consequently preserve adequate function [2]. Today’s treatment options allow this adequate control of disease activity in most of patients [3]. However, there are different definitions of disease activity control. One of these is comprehensive disease control (CDC), which includes several domains related to RA. Achieving CDC means reaching precise targets in tools that assess disease activity, function and radiological damage. Achieving CDC has been shown to provide significant benefits to patients in terms of symptoms (reduced pain and fatigue), quality of life and work ability. The effectiveness of this strategy has been well demonstrated in clinical trials [4, 5]. Achieving CDC is desirable in each RA patient, especially in young patients and in the absence of comorbidity.

However, in everyday clinical practice, the rheumatologist has to deal with patients outside of the “ideal” setting of clinical trials, is increasingly in contact with elderly and comorbid patients and therefore has to relate with subjects at an increased risk of becoming frail. The concept of frailty is relatively recent and emerging in rheumatology. Frailty is a construct aimed at the recognition of individuals who are more vulnerable to adverse events and more predisposed to unfavourable outcomes. The term “frailty” is used to define a category of elderly adults who appear to be vulnerable and weaker than those of the same age with similar demographic characteristics. RA is a disease that predisposes to frailty. The prevalence of frailty in the RA is higher than that of geriatric cohorts: the prevalence is 13% in patients with RA [6], compared to 4–11% in older geriatric cohorts. Pre-frailty conditions are more prevalent in RA patients (69%) than in geriatric cohorts (40–55%) [7, 8].

In the rheumatological literature, there are no data on the impact of frailty in reaching important targets such as CDC. Starting from these considerations, the objective of this study was to verify whether frailty is a factor hindering the achievement of CDC in patients with RA.

## Materials and methods

### Study population and inclusion criteria

Between March 2016 and January 2020, adult patients with RA, as defined by the American College of Rheumatology/European League Against Rheumatism [9], with poorly controlled disease activity (defined by a Simplified Disease Activity Index [SDAI] > 11) with synthetic DMARDs and candidates for treatment with biological DMARDs (bDMARDs) were included in this study. Patients with contraindications to start treatment with bDMARDs, i.e. patients with ongoing infections, active neoplasms, congestive heart failure, pregnant women and patients with comorbidities that may hinder clinical evaluation, i.e. patients with Alzheimer’s disease or other dementias, Parkinson’s disease, ischemic encephalopathy or major depression, were excluded. Once included, the study had an observational character and patients were followed for 1 year after the introduction of bDMARDs in therapy. After 1 year, only patients who maintained treatment with bDMARDs were evaluated, excluding patients who suspended bDMARDs or switched to other bDMARDs or targeted synthetic DMARDs.

### Baseline assessment

Data were collected on demography (age, gender), work activity and education (primary, middle or high school or university). With regard to RA, the duration of the disease (defined by the first swollen joint), the number of tender and swollen joints (on the 28-joint count), visual analogue scale of pain (VAS pain), the physician global assessment of the disease activity (PhGA) and the patient global assessment of disease activity (PaGA) were recorded.

Laboratory indicators included C-reactive protein (CRP), anti-cyclic citrullinated peptide antibodies (ACPA) and rheumatoid factor (RF).

Disease activity was defined by SDAI [10], functional status by the Health Assessment Questionnaire-Disability Index (HAQ-DI) [11], while the presence of frailty was determined by the Comprehensive Rheumatologic Assessment of Frailty (CRAF) [12]. A brief description of these three indices follows.

SDAI employs the algebraic sum of SJC, TJC (28-joint count for these two parameters), PtGA, PhGA and CRP (in mg/dl); values can vary from 0 to 86. A SDAI > 26 defines high disease activity, SDAI > 11 and ≤ 26 defines moderate disease activity, SDAI ≤ 11 and > 3.3 defines low activity and SDAI ≤ 3.3 defines remission [10].

The HAQ-DI evaluates the physical function by estimating the degree of difficulty in performing the activities in eight functional areas, the level of difficulty in the last week is indicated for each activity on a 4-point scale (from 0, no difficulty, to 3, inability to perform). The highest value is considered for each functional area. The final HAQ-DI score is given by the mean of the eight scales [11].

The CRAF is a validated multidimensional index recently developed. CRAF does not require a calculator and investigates 10 health domains (nutritional status, weakness, falls, comorbidity, polypharmacy, social activity, pain, fatigue, physical function and depression). The weight of each factor is attributed according to a predefined table. Among the instrumental equipment for its computation, only a dynamometer is needed to evaluate handgrip strength in the weakness domain [12]. Each domain is given a score of three 0 and 1, then the average of the 10 domains is calculated and the final score ranges from 0 (no deficits present) to 1 (all deficits present). The cut-offs of frailty categories were then defined on the basis of Clegg’s criteria [13]: scores from 0 to 0.12 represent the absence of frailty, scores between 0.12 and 0.24 indicate mild frailty, higher scores between 0.24 and 0.36 indicate moderate frailty, while scores greater than 0.36 indicate patients with severe frailty [12].

The definition of radiological damage used in the CDC calculation was based on the Simple Erosion Narrowing Score (SENS) [14]. X-rays of hands, wrists and feet were then evaluated by a musculoskeletal radiologist (MC) who determined the presence of joint rhyme reduction and the presence of erosion in the joints included in the score. The SENS score ranged from 0 to 86 [14].

Finally, comorbidities were weighed through the Rheumatic Disease Comorbidity Index (RDCI). The RDCI was developed as a tool to assess the effect of comorbidities on quality of life, physical function and costs. The RDCI formula is: 2* lung disease + [2* (myocardial infarction, other cardiovascular diseases or stroke) or 1* hypertension] + 1* (ulcer or other gastrointestinal diseases) + 1* for each of the following conditions: diabetes, fracture, depression and cancer [15].

### Definition of 12-month comprehensive disease control

CDC achievement was evaluated after 12 months of bDMARD treatment. CDC was defined by the contemporary achievement of clinical remission (SDAI < 3.3), normal physical function (HAQ-DI < 0.5), absence of radiographic progression (worsening SENS < 2.28). The score of 2.28 was adopted for SENS because this value represents the smallest detectable change (SDD) for the tool [16].

### Statistical analysis

Data were registered in a Microsoft Excel database and processed with MedCalc 19.0.6 (statistical software packages for Windows XP). The Shapiro-Wilk test was used to study the normal distribution. Data are presented both as mean and standard deviation (SD) and as median and interquartile range (IQR). CRAF scores were calculated and participants were classified as severely frail, moderately frail, mildly frail or non-frail. The comparison between the frailty categories was made using the Mann-Whitney *U* test or the Kruskal-Wallis test for continuous variables and the chi-square test or Fisher's exact test for comparison with the categorical variables. Spearman's non-parametric correlation coefficient was used to evaluate the relationships between clinical, functional and radiological measurements and CRAF scores. Differences in participants’ characteristics between the frailty categories were tested with one-way analysis of variance (ANOVA), or Kruskal-Wallis analysis, where appropriate. *p* values < 0.05 were considered statistically significant. Patients were categorized into CDC achievers vs CDC non-achievers, and the difference between variables (age, gender, educational level, disease duration, BMI, RDCI, RF, ACPA, CRAF and SENS) was firstly assessed with a univariate analysis (Student’s *t* test or Mann-Whitney *U* test). Then, to assess the contribution of the individual independent variables on CDC achievement (dependent variable), a multivariate logistic regression analysis was used.

## Results

A total of 222 RA patients started the study. Eight patients (3.6%) were lost to follow-up and therefore were ruled out. After 1 year, complete data were available for 214 (96.4%) patients (162 women and 52 men), and those were included in the analyses. The mean (SD) age was 60.2 (12.72) years, with a mean (SD) disease duration of 7.34 (2.79) years, and a mean (SD) BMI of 26.39 (4.48). All patients had moderate or active disease, with a mean (SD) SDAI of 27.11 (12.61), and a mean (SD) HAQ-DI of 0.92 (0.54). A total of 149 (69.6%) patients were RF positive and 126 (58.9%) were ACPA positive. Of the 214 patients included, 145 (67.8%) reported one or more medical comorbidities, mostly cardiovascular (26.2%), respiratory (23.4%) and metabolic (22%) disorders. Polypharmacy was very common in our study population, with 57% of the patients receiving 5–8 drugs per day and 13.1% receiving 10 drugs per day or more. The mean number of drugs prescribed per day was 6.2 (SD 2.9, min = 0, max = 14). All patients were receiving at least one bDMARD, respectively, 59 (27.6%) etanercept, 54 (25.2%) adalimumab, 33 (15.4%) abatacept, 26 (12.2%) golimumab, 22 (10.3%) tocilizumab, 11 (5.1%) certolizumab pegol and 9 (4.2%) infliximab. The majority of the patients were on their first biologic agent. Approximately 61% of the patients receive a csDMARD, usually methotrexate (72.3%). A total of 102 patients (47.7%) were taking oral corticosteroids, at a mean prednisone or equivalent dose of 3.9 mg/day (range 2.5–16), and 131 (61.2%) were prescribed non-steroidal anti-inflammatory drugs (NSAIDs) on demand. Table 1 summarizes the baseline characteristics of the patients.

**Fig. 1:**
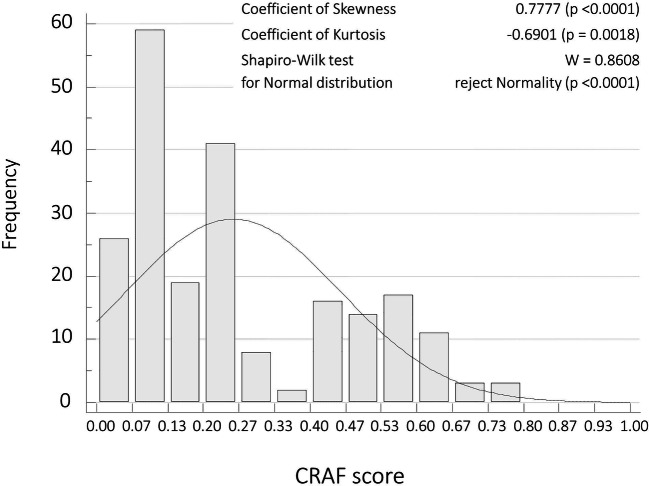
Comprehensive Rheumatologic Assessment of Frailty (CRAF) scores distribution in the patients’ cohort (214 patients)

Figure 1 presents estimates of central tendency and distribution of the CRAF, which was non-normally distributed. The median of CRAF was 0.20 (IQR, 0.08–0.43) (Table 1), and according to CRAF definition, 84 (39.3 %) patients were non-frail, 57 (26.6%) were mildly frail, 14 (6.5%) were moderately frail and 59 (27.6%) were severely frail.

**Table 1:** Baseline demographic, laboratory and clinimetric data of the cohort (214 patients).

	Mean	Median	SD	25–75 P
Age (years)	60.25	60.00	12.72	50.00–70.00
Disease duration (years)	7.34	7.00	2.59	5.00–10.00
Educational level (years)	11.17	13.00	4.20	8.00–13.00
BMI (kg/m^2^)	26.39	4.48	25.52	23.03–28.71
RDCI (score, 0–11)	1.90	1.00	2.01	0.00–3.00
ACPA (titre, UI/ml)	232.46	120.00	383.31	10.00–309.00
RF (titre, UI/ml)	141.92	55.60	209.95	10.00–151.00
HAQ-DI (score, 0–3)	0.92	0.92	0.42	0.62–1.12
SDAI (score, 0–86)	27.09	25.24	12.49	20.32–36.25
SENS (score, 0–78)	11.71	12.00	7.19	5.00–17.00
CRAF (score, 0–10)	0.25	0.20	0.20	0.08–0.43

*SD* standard deviation, *P* percentiles, *BMI* body mass index, *RDCI* Rheumatic Diseases Comorbidity Index, *HAQ-DI* Health Assessment Questionnaires Disability Index, *ACPA* anti-cyclic citrullinated peptide antibodies, *RF* rheumatoid factor, *SDAI* Simplified Disease Activity Index, *SENS* Simple Erosion Narrowing Score, *CRAF* Comprehensive Rheumatologic Assessment of Frailty

**Fig. 2:**
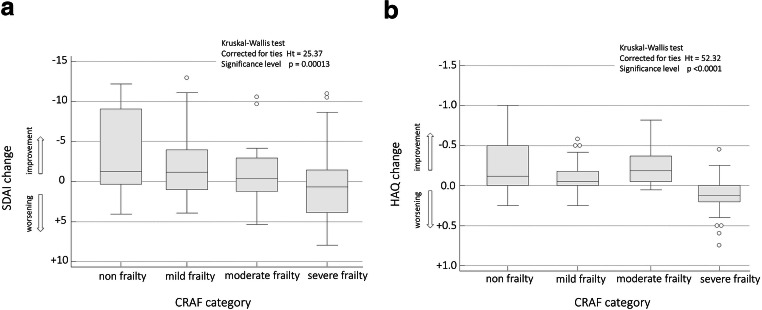
Disease activity and functional ability according to frailty categories. Box–whisker plots showing the changes in the scores of Simplified Disease Activity Index (SDAI) (A) and of Health Assessment Questionnaire Disability Index (HAQ-DI) (B) after 12 months, in relation to the frailty categories (Kruskal-Wallis test) of the Comprehensive Rheumatologic Assessment of Frailty (CRAF). The horizontal lines in each box represent the median, and the box height represents the interquartile range

Distinguishing patients according to the CRAF category, after 12 months of treatment, the disease activity (Fig. 2a) and function (Fig. 2b) showed a significant improvement in non-frail patients compared to severely frail patients (*p* = 0.00013 for SDAI, *p* < 0.0001 for HAQ-DI). No significant changes were also identified with respect to the structural damage measured with SENS (*p* = 0.092).

At 12 months, CDC was achieved in 31 (14.4%) patients versus 183 (85.6%) patients who did not achieve CDC. Table 2 summarizes the differences at baseline between variables in CDC achievers vs CDC non-achievers in the univariate analysis. Statistically significant differences emerged in all variables, which were subsequently included in the logistic regression analysis. The variables associated with CDC achievement in logistic regression analysis are presented in Table 3. The CRAF score was found to be the only independent variable significantly related (*p* = 0.004) to CDC achievement.

**Table 2:** Univariate analysis (Student’s *t* test or Mann-Whitney *U* test) to evaluate the differences in demographic and clinical variables between CDC achievers vs CDC non-achievers

	CDC non-achievers (no 183)				CDC achievers (no 31)				
	Mean	Median	SD	25–75 P	Mean	Median	SD	25–75 P	p
Age (years)	63.98	64.00	12.59	57.00–72.00	56.38	52.00	11.40	44.00–60.00	0.022
Disease duration (years)	8.78	7.00	2.63	5.00–10.00	6.07	6.00	2.23	5.00–8.00	0.038
Educational level (years)	10.03	13.00	3.86	8.00–13.00	13.02	15.00	2.38	13.00–16.00	0.048
RDCI	2.53	1.00	2.09	0.25–3.00	1.52	1.00	1.45	0.00–2.00	0.003
BMI (k/m^2^)	27.33	27.00	4.32	24.80–29.50	25.09	25.00	3.98	22.50–28.00	0.048
RF (titre)	188.82	53.30	212.40	10.00–149.25	134.89	82.00	206.44	11.25–309.00	0.036
ACPA (titre)	249.89	122.00	399.21	10.00–309.00	219.89	120.00	308.85	55.15–339.75	0.044
CRAF (score)	0.29	0.22	0.20	0.08–0.47	0.20	0.08	0.11	0.07–0.09	0.033
SENS (score)	10.57	12.00	7.05	5.00–16.75	12.38	14.00	7.88	5.25–18.00	0.039

*CDC* comprehensive disease control, *P* percentiles, *BMI* body mass index, *RDCI* Rheumatic Diseases Comorbidity Index, *HAQ-DI* Health Assessment Questionnaires Disability Index, *RF* rheumatoid factor, *ACPA* anti-cyclic citrullinated peptide antibodies, *SDAI* Simplified Disease Activity Index, *SENS* Simple Erosion Narrowing Score, *CRAF* Comprehensive Rheumatologic Assessment of Frailty

**Table 3:** Logistic regression analysis showing the baseline independent variables associated with comprehensive disease control achievement (dependent variable) after 12 months

Independent variables	Coefficient	Standard error	Wald	Odds ratio	95% CI	*p*
Age (years)	0.0089	0.0235	0.1443	1.0003	0.95–1.04	0.7041
Gender	0.7603	0.5233	2.1105	2.1390	0.76–5.96	0.1463
Educational level (years)	0.0949	0.0571	2.7570	1.0996	0.98–1.23	0.0968
Disease duration (years)	-0.0997	0.0862	1.3388	0.9050	0.76–1.07	0.2472
RDCI (score)	0.2174	0.1992	1.1908	1.2429	0.84–1.83	0.2752
BMI (score)	0.0978	0.0556	2.8090	0.8970	0.76–1.08	0.0995
RF (titre)	0.0004	0.0008	0.2818	1.0005	0.99–1.00	0.5955
ACPA (titre)	0.0002	0.0005	0.2058	1.0003	0.99–1.00	0.6501
CRAF (score)	− 7.4985	2.6069	8.2735	0.0001	0.00–0.02	0.0040
SENS (score)	0.0007	0.0005	0.8667	1.0891	0.96–1.29	0.2449
Constant	− 1.7101	1.6833	1.0320			0.3097

*CI* confidence intervals, *RF* rheumatoid factor, *ACPA* anti-cyclic citrullinated peptide antibodies, *CRAF* Comprehensive Rheumatologic Assessment of Frailty, *SENS* Simple Erosion Narrowing Score

Table 4 shows the mean (SD) and median (IQR) change scores of SDAI, HAQ-DI and SENS at 12-month intervals in CDC achievers and in non-achievers. CDC achievers had significantly (*p* < 0.001) lower changes or worsening, in physical function (HAQ-DI) and disease activity (SDAI). No statistically significant changes (*p* = 0.092) were found in radiologic damage (SENS) between the two groups.

**Table 4:** Relationship between the comprehensive disease control (CDC) achievers and non-achievers over 12 months changes of the patient disease activity (SDAI), functional (HAQ-DI) and structural (SENS) variables

	CDC achiever (31 patients)				CDC non-achiever (183 patients)			
	Mean	Median	SD	25–75 P	Mean	Median	SD	25–75 P
SDAI change	0.62	0.45	3.65	− 1.33–2.15	8.93	9.78	3.68	9.04–10.53
HAQ-DI change	0.015	0.00	0.21	− 0.12–0.12	0.58	0.60	0.32	0.50–0.73
SENS change	3.29	2.00	3.27	0.00–6.00	1.58	1.00	1.70	0.00–3.75

*CDC* comprehensive disease control, *SD* standard deviation, *P* percentiles, *SDAI* Simplified Disease Activity Index, *HAQ-DI* Health Assessment Questionnaires Disability Index, *SENS* Simple Erosion Narrowing Score

## Discussion

This is the first study demonstrating that frailty is a condition that reduces the chances of achieving CDC in RA patients.

Achieving complete control of RA in terms of disease activity, function and joint damage is the ideal goal in all patients. Identifying the factors that hinder the achievement of this goal is a prerogative for the rheumatologist [17, 18].

Frailty is a syndrome with multiple causes contributing to its onset. Frailty is characterized by a reduction of strength, resistance and physiological functions, predisposing the subject to be more vulnerable and at greater risk of becoming dependent or dying [19–21]. Among its clinical expressiveness, RA can predispose patients to multiple factors included in the frailty definition [21].

 Well-known in the geriatric field, where it has been documented that it is associated with multiple unfavourable outcomes (risk of hospitalization, institutionalization and death) [22, 23], frailty must also be recognized in other settings such as rheumatology since it is an evolutive but potentially reversible syndromic scenario [24, 25].

The prevalence of frailty (defined as severe frailty at CRAF) in our cohort of RA patients, with a mean age of 60 years, was 27.6%. This prevalence is significantly higher than that described in cohorts 10 years older. Pre-frailty conditions (mild and moderate frailty at CRAF) are in line with those of the geriatric cohorts [7, 8]. Compared to other cohorts of RA patients, who used the definition of the Survey of Health, Aging and Retirement in Europe–Frailty Instrument (SHARE-FI) [26], the prevalence observed in our study was higher. This may be due to the fact that SHARE-FI mainly evaluates frailty limited to a biopsychosocial paradigm. SHARE-FI is devoted to primary care; it does not take into account variables such as pain, fatigue and depression, very common conditions in RA patients [26].

The interrelationships between inflammation, physical fatigue, muscle dysfunction, pain and psychological factors have been suggested as implied pathogenic mechanisms of frailty in RA patients [27]. However, the specific mechanisms of frailty in RA have not been studied in detail. Previous studies demonstrated that a high disease activity, assessed by SDAI, was highly correlated to frailty [12, 28]. Systemic inflammation is closely linked to frailty [29]. RA is also characterized by early ageing of the immune system [30–32]. High levels of IL-6 and TNFα are correlated with reduced muscle mass and reduced strength [33, 34]. Frailty, reduced functional ability and reduced mobility are associated with higher levels of pro-inflammatory cytokines [35, 36]. Some experimental evidence also documents the direct action of certain cytokines on the central nervous system in favour of the perception of fatigue [37, 38]. Fatigue can be considered a manifestation of changes in neuronal function secondary to inflammatory stimuli [39]. IL-6, the pro-inflammatory cytokine by definition, can cross the blood-brain barrier and induce these neuronal changes.

Frailty is also predicted by pain, emphasizing the importance of its treatment, potentially contributing to the prevention of vulnerability, dependency and mortality [40]. Among comorbidities inducing frailty, depression is the most frequent in RA patients. A 2013 systematic review and meta-analysis found that 16.7% of those with RA meet diagnostic criteria for major depressive disorder [41]*.* Comorbid depression negatively impacts RA patients’ health-related quality of life, physical function, mental function, mortality and experience of pain and symptom severity [42]. Depressive symptoms may be a risk factor for frailty, causing changes in behaviour and activity that result in increased disability, leading to frailty [43]. Conversely, depressive symptoms may also be an early symptom of frailty. Sanders et al. demonstrates a strong association between pain and depressive symptoms over time [44]. Moreover, this association remained unaffected by follow-up time, age or frailty status. This interaction has been labelled by some as the depression-pain dyad [45]. With an increasing number of comorbidities occurring with ageing, a better understanding of the reciprocal link between pain and depression may identify factors suitable for prevention or improved treatment outcomes.

Pain and depression are other frailty-related variable. Of fundamental importance is the treatment of pain, which could contribute to avoid the onset of frailty itself and its consequences [40]. A detailed understanding of the interaction between pain and depression can lead to the identification of factors on which preventive interventions can be implemented.

Several limitations of our study should be recognized. First, since our main objective was to determine if frailty was associated with a therapeutic response, the analysis was limited to a time interval. Secondly, certain CRAF parameters, such as depression, were detected by a VAS in the thermometer format [46]. This type of patient-reported method is certainly not the optimal one. Third, the effect of other factors, such as the use of other drugs (e.g. corticosteroids), was not investigated. Fourth, although the components of CDC have been found in clinical trials, some variables, such as HAQ-DI, may be influenced by age, and over 65 years of age an HAQ-DI < 0.5 may be incorrect as a functional remission criterion [47]. Finally, few patients reached CDC at 12 months, so the analyses may have been affected by the small number of patients in this subgroup.

The main strength of this study is the prospective observational design, and the novelty of using a dedicated frailty index should be emphasized. The results of this study suggest dedicating more and more attention to frailty in patients with RA, in all its determinant variables, through dedicated tools such as CRAF or other validated frailty questionnaires [48].

## Data Availability

The data are available upon reasonable request to the corresponding author.
